# Qbd-Based Approach to Optimize Niosomal Gel of Levosulpiride for Transdermal Drug Delivery

**DOI:** 10.3390/gels9030213

**Published:** 2023-03-10

**Authors:** Ahmed S. Alnaim, Hiral Shah, Anroop B. Nair, Vivek Mewada, Smit Patel, Shery Jacob, Bandar Aldhubiab, Mohamed A. Morsy, Rashed M. Almuqbil, Pottathil Shinu, Jigar Shah

**Affiliations:** 1 Department of Pharmaceutical Sciences, College of Clinical Pharmacy, King Faisal University, Al-Ahsa 31982, Saudi Arabia; 2 Department of Pharmaceutics, Arihant School of Pharmacy & BRI, Adalaj, Gandhinagar 382421, India; 3 Department of Pharmaceutics, Institute of Pharmacy, Nirma University, Ahmedabad 382481, India; 4 Department of Pharmaceutical Sciences, College of Pharmacy, Gulf Medical University, Ajman 4184, United Arab Emirates; 5 Department of Pharmacology, Faculty of Medicine, Minia University, El-Minia 61511, Egypt; 6 Department of Biomedical Sciences, College of Clinical Pharmacy, King Faisal University, Al-Ahsa 31982, Saudi Arabia

**Keywords:** levosulpiride, niosomes, Box-Behnken design, evaluation, transdermal, pharmacokinetics

## Abstract

Poor aqueous solubility besides extensive hepatic first effect significantly decreases the oral absorption of levosulpiride, which in turn minimizes its therapeutic effectiveness. Niosomes have been extensively investigated as a transdermal vesicular nanocarrier to increase the delivery of low permeable compounds into and across the skin. This research work was to design, develop and optimize levosulpiride-loaded niosomal gel and to evaluate its prospects for transdermal delivery. The Box-Behnken design was used to optimize niosomes by analyzing the impact of three factors (cholesterol; X_1_, Span 40; X_2_, and sonication time; X_3_) on the responses (particle size, Y_1_, and entrapment efficiency, Y_2_). Optimized formulation (NC) was incorporated into gel and evaluated for pharmaceutical properties, drug release study, ex vivo permeation, and in vivo absorption. The design experiment data suggest that all three independent variables influence both response variables significantly (*p* < 0.01). Pharmaceutical characteristics of NC vesicles showed the absence of drug excipient interaction, nanosize (~102.2 nm), narrow distribution (~0.218), adequate zeta potential (−49.9 mV), and spherical shape, which are suitable for transdermal therapy. The levosulpiride release rates varied significantly (*p* < 0.01) between niosomal gel formulation and control. Greater flux (*p* < 0.01) was observed with levosulpiride-loaded niosomal gel than with control gel formulation. Indeed, the drug plasma profile of niosomal gel was significantly higher (*p* < 0.005), with ~3 folds higher C_max_ and greater bioavailability (~500% higher; *p* < 0.0001) than its counterpart. Overall, these findings imply that the use of an optimized niosomal gel formulation can increase the therapeutic efficacy of levosulpiride and may represent a promising alternative to conventional therapy.

## 1. Introduction

Transdermal drug administration through the skin is challenging due to multiple physical, chemical, and biological barriers. The main rate-limiting barrier of the skin, the stratum corneum, is an uphill task for transdermal drug delivery scientists [1]. Researchers have explored a variety of cutting-edge approaches to solve this problem and continue to work on it to find an effective solution to the present day [2,3,4,5,6]. In the last few decades, the usage of nanovesicular carriers as a drug delivery system for drug transportation through the skin has received many fruitful results [2,7,8]. This is primarily due to the potential of vesicular systems to significantly change the physicochemical properties of the drug when encapsulated and supports the percutaneous absorption of large hydrophilic, hydrophobic, or uncharged molecules [9].

Vesicular systems, such as liposomes, have problems with stability, which ultimately leads to drug leakage. On the other hand, non-ionic surfactant-containing niosomal vesicular systems could be a different type of carrier system for transdermal drug administration [10]. These systems have several advantages over liposomal systems, including versatility, great penetration potential, excellent physical and chemical stability, lower production cost, reduced toxicity, ease to be formulated and scale-up, biocompatibility, biodegradability, and non-immunogenicity [11,12]. Several studies have demonstrated the ability of niosomes to transport drug molecules through intact skin, indicating the potential of this carrier in transdermal therapy [13,14].

The gel-based transdermal therapy has proven to be more efficient compared to other transdermal drug delivery systems [15,16]. This is because the gel composition offers better patient compliance, less tackiness, greater therapeutic effectiveness, increased residence time, rapid drug release, customized pharmacokinetics, and almost no or minimal skin irritation [17,18,19,20,21,22]. Meantime, understanding the drug’s physicochemical properties and its therapeutic efficacy in addition to the functionality of excipients are key factors in the selection of a transdermal system [16,23].

Levosulpiride, a hydrophobic sulpiride moiety, a derivative of benzamide used as a neuroleptic and prokinetic compound for the therapy of various CNS diseases like depression, psychosis, somatoform disorders, emesis, and dyspepsia [24]. It is a selective dopamine D2 antagonist, that blocks dopamine secretion from the receptor [25]. Moreover, levosulpiride proved its effectiveness clinically in the treatment of patients with various conditions at reduced doses [26,27]. Despite its clinical significance, levosulpiride is a BCS class IV drug with poor solubility and bioavailability (<30%) and extensive first-pass effect, which poses a significant challenge in its development as an oral dosage formulation [28]. The objective of this research was to develop and optimize levosulpiride-loaded niosomal gel using a Box-Behnken design to enhance the percutaneous absorption and also to check its potential in a rat model. Optimization studies were performed by checking the independent variables such as concentration of formulation components (cholesterol and Span 40) and process variable (sonication time) on evaluation parameters like the size of niosomes particles as well as entrapment efficiency. The optimized niosomal gel formulation was assessed for its percutaneous absorption by ex vivo studies and further evaluated in vivo in rats by assessing pharmacokinetic parameters against the control gel. 

## 2. Results and Discussion

### 2.1. Results of Preliminary Trials for Selection of Excipients

The selection of niosomes components is critical as they play an important role and have a huge influence on pharmaceutical properties as well as in vivo performance [29]. Hence, the preliminary study evaluated the selection of niosomes components by assessing their EE, which indicates the extent of payload or drug retention capacity [30]. Prepared preliminary formulations were estimated for % EE and the results are summarized in Table S1. Maximum EE (64.21%) was observed in the P5 batch, which was prepared by using Span 40: cholesterol (1:1 mM) and a sonication time of 10 min. Based on the results, Span 40 (non-ionic surfactant) was chosen for future studies. Indeed, Span 40 has been extensively used in the transdermal delivery of various drug molecules, according to the literature [31]. Moreover, the nanocarriers formulated using Spans demonstrated good stability and were less leaky [14]. Literature suggests that the amount of cholesterol and non-ionic surfactants influence the particle size and EE of niosomes [32,33,34,35]. Thus, these three factors and their levels were selected as independent variables for further optimization study.

### 2.2. Optimization of Variables Using Box-Behnken Design

The use of Design of Experiment (DoE) tools in optimizing nanoformulations like niosomes has numerous benefits, including higher product quality and efficacy [36]. Design batches (N1–N15) were prepared and various pharmaceutical parameters were evaluated. The particle size (hydrodynamic diameter in nm) and EE (%) for the designed formulations were considered as responses and the results are shown in Table 1. The measured responses of design batches were tailored to the quadratic model. The equations derived for both responses were transformed, interpreted, and used to conclude the results. 

### 2.3. Data Analysis for Dependent Variables of Design Batches

#### 2.3.1. Particle Size 

Particle size is an important factor in characterizing niosomes because it is related to formulation efficiency, particularly in transdermal therapy [37]. The designed batches of the levosulpiride-loaded niosomes formulations achieved hydrodynamic diameters in the range from 72 nm to 202 nm. This wide range in particle size suggests that the variables have an impact on the size of the formulated niosomes. According to the results, the quadratic equation was derived using multiple regression analysis;

Y1= 199.50 + 61.61X_1_ − 2.50X_2_ − 2.64X_3_ − 0.8500X_1_X_2_ − 0.7250X_1_X_3_ − 34.59X_1_^2^ − 28.26X_2_^2^ − 27.79X_3_^2^

**Figure 1:**
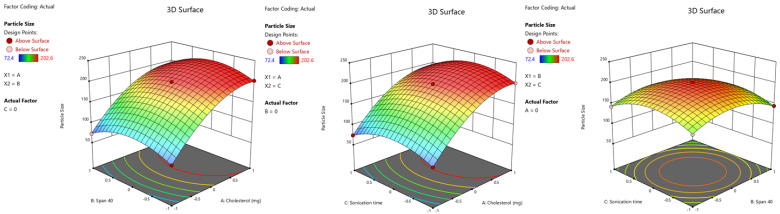
3D surface response plots of the effect of factors on particle size.

This analysis shows the model is significant (F value = 3087.23, *p* < 0.0001, and R^2^ value = 0.999). The cholesterol amount (X_1_) showed a noticeable positive result (*p* < 0.0001, b = +61.6) for niosomes particle size, which signifies that an increase in cholesterol amount could lead to a higher particle size of niosomes, in the current experimental conditions. These results were also consistent with the earlier studies reported [38,39], which show that increasing the cholesterol amount, makes the membrane more rigid hence reducing the effect of sonication and can produce large-size niosomes. It is also described in the literature that the cholesterol changes the fluidity of the chains in the bilayers and hence the breadth of the lipid layer which in turn leads to large niosomes [40]. On the other hand, the amount of Span 40 (X_2_) and sonication time (X_3_) demonstrated a negative effect on the size of prepared niosomes and were significant (*p* < 0.001). The minus sign noticed in Span 40 indicates that a minor increase in the non-ionic surfactant level will lead to a reduction in niosomes size, which might be due to a drop in the surface energy, ultimately producing small-size niosomes. Similarly, the increase in sonication time leads to a decrease in niosomes size. The *p*-value for the individual coefficients in the model is shown in the ANOVA table (Supplementary Table S2). The influence of cholesterol, Span 40, and sonication time on niosomes size as a 3D surface response plot is shown in Figure 1.

#### 2.3.2. %. EE

The % EE indicates the amount of drug molecules that were successfully entrapped inside the niosomes. The type of non-ionic surfactant, formulation method, and ingredients used could have a significant impact on the EE of niosomes [29]. The % EE of the designed niosomes formulation ranged from 51% to 88%, which indicates that the independent variables have an impact on the EE of the niosomes. The equation related to EE was shown below;

Y_2_ = 74.49 + 14.47X_1_ − 4.59X_2_ − 0.0650X_3_ + 2.84X_1_X_2_ − 0.1425X_1_X_3_ − 0.3275 X_2_X_3_ − 1.31X_1_^2^ − 0.2921X_2_^2^ + 0.9079X_3_^2^

**Figure 2:**
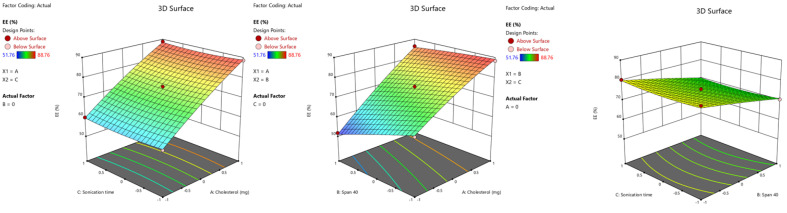
3D surface response plots of the effect of independent variables on EE (%).

The model for the entrapment efficacy is significant [F value = 147.33 (*p* < 0.0001), and the R^2^ value is 0.9962]. From the above equation, it is shown that the positive coefficient of the cholesterol amount (X_1_) (b = +14.47) demonstrated a significant (*p* < 0.0001) impact on the entrapment efficacy. This is most likely due to an increase in the amount of cholesterol causing an improvement in bilayer hydrophobicity and formation of a more rigid membrane [41,42], which may aid in the efficient entrapment of levosulpiride inside the vesicles. Thus, an increase in cholesterol amount enhances the drug entrapment efficacy, as well as increases the size of the niosomes. The Span 40 (X_2_) demonstrated a significant negative (*p* < 0.0001, b = −4.59) impact on EE. This is probably due to the micellar solubilization of levosulpiride in aqueous media, as well as its binding with Span 40 on the surface of niosomes, which can lead to less drug entrapment inside niosomes. Equally, the increase in sonication time leads to a decrease in the niosome’s size. The *p*-value for the individual coefficients in the model is shown in the ANOVA table (Supplementary Table S3). The effect of cholesterol, Span 40, and sonication time on EE as a 3D surface response plot is shown in Figure 2.

Assessment of possible correlation between responses (particle size and % EE) indicates a poor relationship (r^2^ = 0.716). This is likely because the formulation components, particularly cholesterol, have a substantial impact on both responses.

### 2.4. Optimization and Validation of Response Surface Analysis 

According to the statistical analysis and graphical presentation of the impact of the factors on the responses, the optimized level of X_1_, X_2,_ and X_3_ were identified on the overlay plot (Design Expert), which could contribute to achieving the target responses. It is well known that the niosomes with small sizes will enhance permeation when applied on the skin surface [43,44]. Hence, niosomes formed with low particle size could be ideal for the transdermal delivery of levosulpiride. 

**Figure 3:**
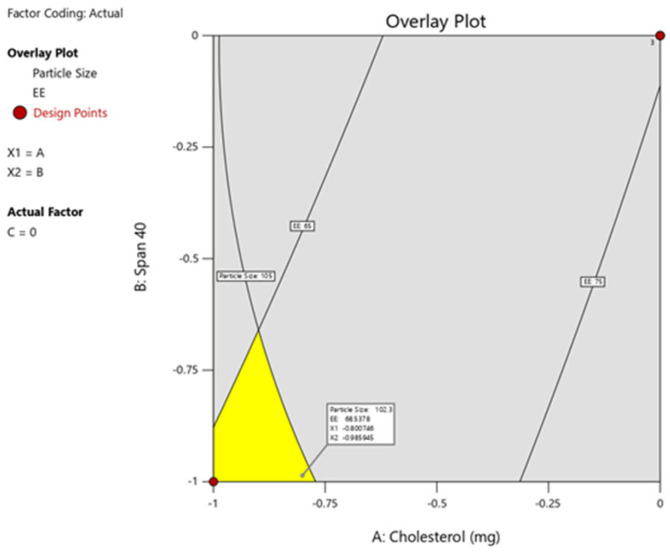
Software-generated overlay plot for the selection of the best batch from design space.

The overlay plot (Figure 3) was utilized to validate the model by comparing the predicted values (Y_1_ = 102.3 nm and Y_2_ = 68.537%) with practically obtained values (Y_1_ = 102.2 nm and Y_2_ = 67.98%). 

#### Particle Size and Zeta Potential of Optimized Batch

**Figure 4:**
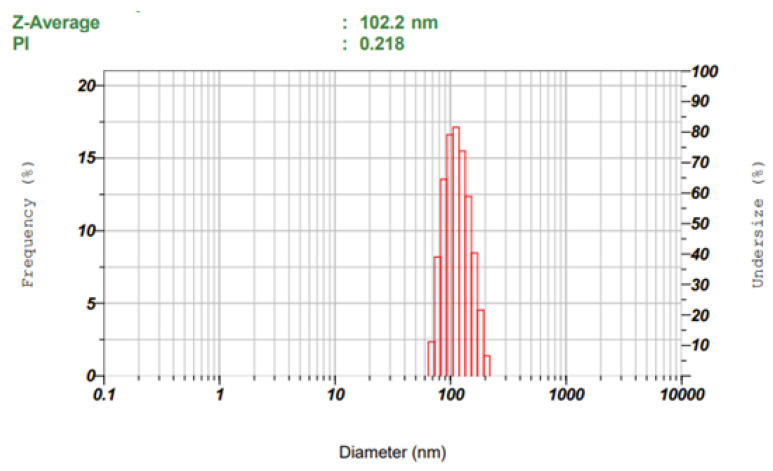
Particle size distribution of optimized batch (NC).

**Figure 5:**
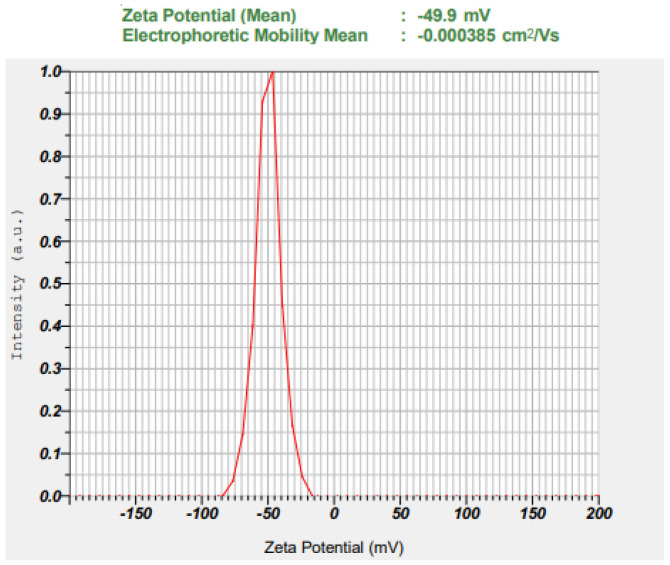
Zeta potential of optimized batch (NC).

Based on a software-generated overlay plot, the batch was prepared and both particle size and zeta potential were measured. After check point analysis, this batch was considered an optimized batch (NC). A graph of the size distribution of the optimized niosomes was illustrated in Figure 4, which shows an average particle size of 102.2 ± 5.4 nm with narrow size distribution. Figure 5 displays the zeta potential distribution of the optimized batch, and the value recorded was −49.9 ± 7.1 mV, which is >± 30 mV, signifying higher stability of the prepared niosomes [45]. The zeta potential values observed here are high enough for the required repulsion between the particles and for the electrostatic stabilization of niosomes. 

### 2.5. Check Point Analysis

Table 2 shows the comparison of projected and observed values of check point formulation (NC). Results show the insignificant difference observed between values for particle size and % EE. Therefore, the design model selected for the study is validated. 

### 2.6. FTIR

**Figure 6:**
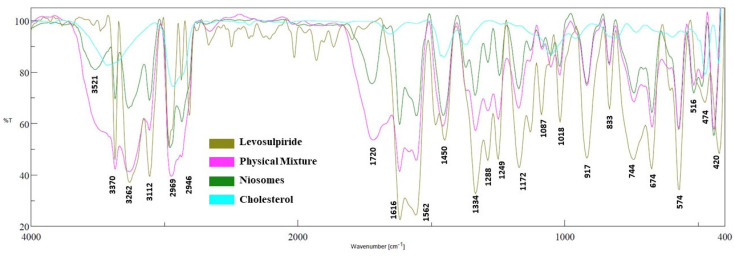
FTIR spectra of levosulpiride, physical mixture, cholesterol, and niosomes formulation.

FTIR spectroscopy study was done to check the possible incompatibilities of levosulpiride with additives used in the optimized niosomes. The spectra of various samples like levosulpiride, cholesterol, physical mixture, and optimized batch are depicted in Figure 6. The drug has shown absorption peaks because of –N–H stretching (3370.96 cm^−1^), N–H bending (3266.82 cm^−1^), O–H bending (3112.55 cm^−1^), pyrrolidine stretch (2969.84 cm^−1^), %#x2013;C–H stretching (2873.42 cm^−1^), –C=O stretching (1616.06 cm^−1^), C–C stretching (1558.20 cm^−1^), aromatics –C=C stretching (1450.21 cm^−1^), strong sulphonyl absorption (1334.50 cm^−1^), and C–N stretching (1087.66 cm^−1^) as reported elsewhere [24]. The spectra of the physical mixture reveal the main and specific peaks of the drug and excipients. The FTIR spectra of niosomes reveal the drug and excipient’s unique absorption peaks at the same position as peaks observed in the spectra of levosulpiride. Moreover, the presence of all drug peaks in the range of 2900–3400 cm^−1^ in niosomes further confirms the absence of incompatibility of the drug with organic solvents and surfactants used. Overall, the result suggests the formulation ingredients used here are compatible with levosulpiride.

### 2.7. DSC

**Figure 7:**
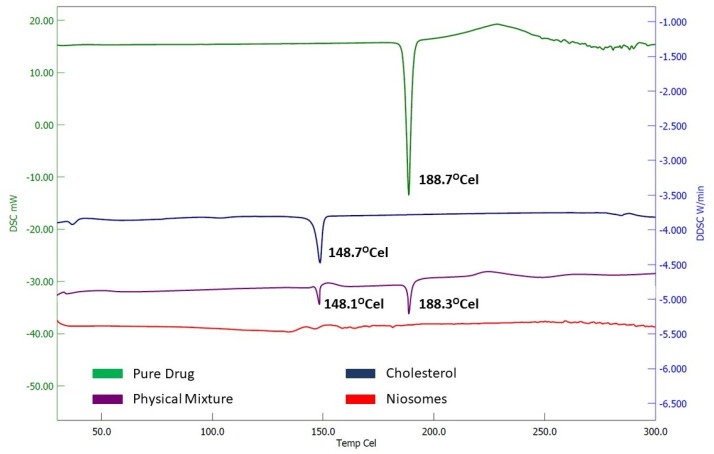
Differential scanning calorimetric graph of levosulpiride, cholesterol, physical mixture, and niosomes formulation (NC).

DSC is used to understand the thermal behavior of drugs and other components used in the niosomes as well as detect phase transitions like melting and crystallization. The DSC thermogram (Figure 7) revealed sharp endothermic peaks at 188.7 °C and 148.7 °C, which represents the corresponding melting temperature of levosulpiride and cholesterol, respectively. The DSC of the physical mixture shows typical peaks of the drug and cholesterol with a reduced intensity, while the niosomes formulation shows no characteristic peak of the drug, which may be due to the complete solubilization of the levosulpiride within the niosomal structure. 

### 2.8. TEM

**Figure 8:**
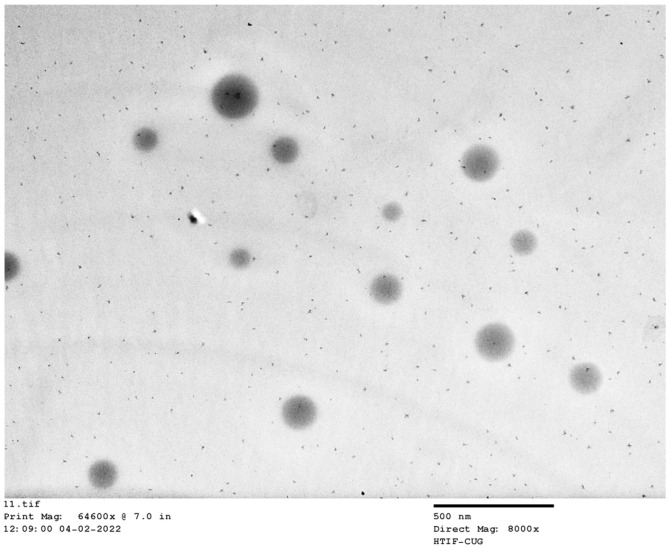
A representative transmission electron microscopy image of levosulpiride niosomes (NC).

TEM is an important technique for obtaining high-resolution images of nanocarriers with morphological characteristics such as size/shape [46]. A representative TEM image of the optimized levosulpiride niosomes formulation (NC) was depicted in Figure 8. It is apparent from the figure that the niosomes vesicles are symmetric and separated. In addition, the image also suggests the prepared particles seem to be closed, circular in shape, and of nanometer size. Indeed, the particle size (range of 50–200 nm) depicted in Figure 8 matched the actual values observed during the size measurement in Figure 4.

### 2.9. Evaluation of Levosulpiride-Niosomal Gel

The physicochemical characteristics of topical gel are of prime importance as they could affect the efficacy of the formulation [47]. The drug content was observed between 90–95% in prepared formulations. The pH of the gel composition was in the range of 6.5–7 and did not vary between the formulations. The rheological characteristics, like viscosity and spreadability, are well connected with each other and also play an effective role in a gel formulation, for its strength as well as its efficacy. The viscosity of optimized niosomes gel at 25 ± 1.0 °C (8445 ± 103 cPs) and 32 ± 1.0 °C (8370 ± 96 cPs) varied minimally. The viscosity observed here is comparable to the previously reported values [48]. 

**Figure 9:**
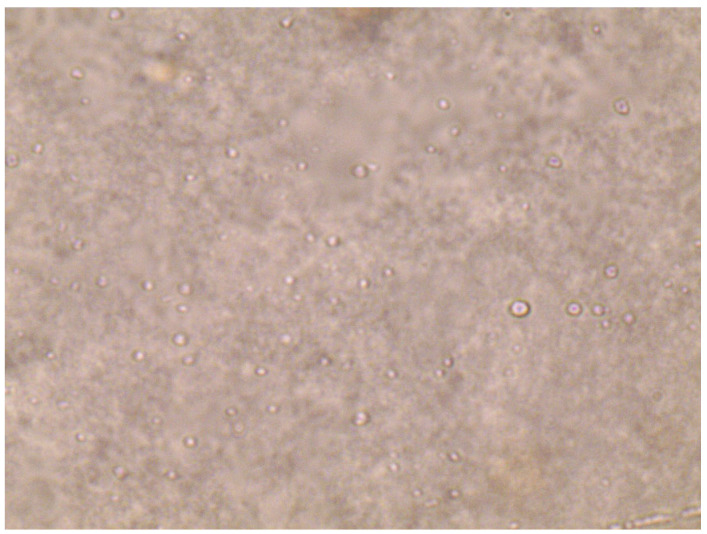
A representative light microscopy picture of levosulpiride niosomal gel.

The microscopic image (Figure 9) of the niosomal gel displayed the uniform distribution of spherical niosomes in the gel matrix. Adhesion refers to the bonding of gel on the skin membrane with a short contact time when light pressure is applied. In the current study, the maximum force at which the gel detaches was measured as an adhesive force. The hardness value indicates the force necessary for required deformation. The observed values for the gel adhesion and hardness were 128.10 g*s and 176 g, respectively (Figure S1).

### 2.10. In Vitro Release Study

**Figure 10:**
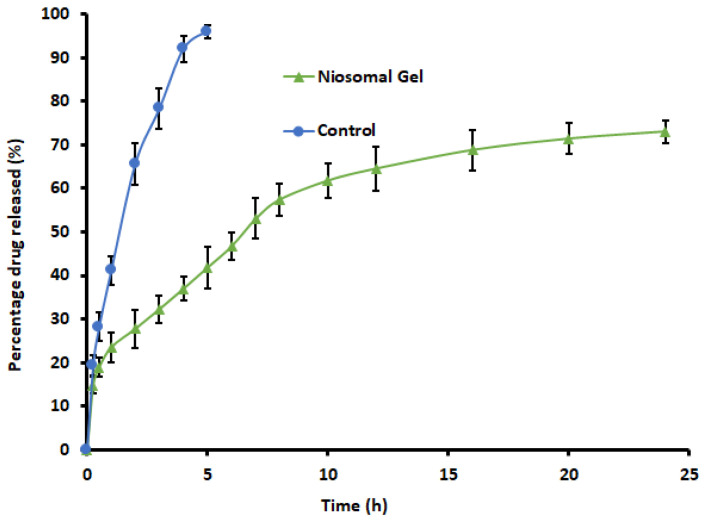
Comparison of release profiles of levosulpiride from niosomal gel and control gel. Each value depicts the average and standard deviation of six different batches.

The in vitro release profile provides significant insight into formulation structure and behavior, potential drug-carrier interactions, and the impact of these on the mechanism and rate of drug release. The comparison of in vitro drug release of levosulpiride-loaded niosomes gel and control was depicted in Figure 10. It can be seen from the figure that the release profiles of both the drug-loaded niosomal gel formulation and the control were different (*p* < 0.01). The levosulpiride release from optimized niosomes was rapid (~15%) in 0.25 h and then became linear for the next period (0.25–8 h) with ~57% being released. This is followed by a gradual release of levosulpiride in a concentration-dependent manner for the remaining study period (up to 24 h). The rapid release could be attributed to the loosely bound drug, whereas the linear/sustained release observed could be attributed to the release retarding effect of bilayers, which allowed the payload to diffuse out slowly, as reported in the literature [47]. Indeed, this type of profile is significant because the ideal niosomal gel should have prolonged drug release to reduce the need for repeated application [49]. On the other hand, the drug release from the control gel was rapid and almost complete in 5 h, which signifies the drug can freely pass through the cellulose membrane used. In vitro release pattern of levosulpiride from the prepared gel was scientifically examined to explain the kinetics. Analysis of the release mechanism reveals the profile of levosulpiride from optimized niosomes gels is linear with the Korsmeyer–Peppas model with a high correlation coefficient (r^2^ = 0.9813). The n value of 0.3772 noticed suggests the kinetic pattern is Fickian diffusion, which is often observed with niosomes [50,51].

### 2.11. Ex Vivo Permeation Study

**Figure 11:**
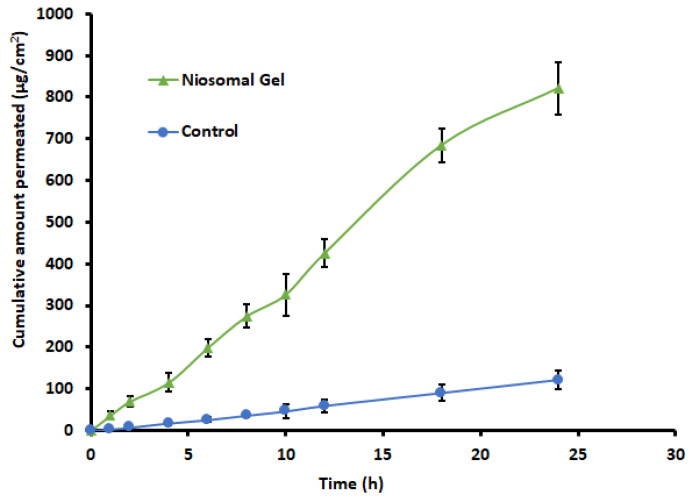
Ex vivo permeation profiles of levosulpiride from niosomal gel and control gel across rat skin. Each value depicts the mean and standard deviation of six different batches.

Permeation studies are typically performed to forecast the drug transport across the skin during their in vivo application. The physical and chemical characteristics of the permeant, the physiological characteristics of the barrier, such as the thickness and composition of the membrane, and the availability of a transport route for drug permeation will all influence transdermal drug delivery [52,53]. The comparison of ex vivo permeation data of levosulpiride-containing niosomes gel and control gel is shown in Figure 11. The profiles show that levosulpiride permeation from niosomes was significantly higher (*p* < 0.01) than the control. The cumulative amount of drug permeation steadily increases with duration in the case of niosomes, unlike the control gel where it was relatively low. The average steady-state flux by niosomes (35.82 µg/cm^2^/h) and control (5.15 µg/cm^2^/h) indicates a sevenfold increase in the drug transport with levosulpiride-loaded niosomes. The low flux value of levosulpiride notices with control could be correlated to its BCS classification IV. The lag time found with niosomes and control gel was 0.29 and 0.64 h, respectively, which indicates that the drug-loaded niosomes quickly penetrate the skin. The literature describes many mechanisms that may help to improve niosomes penetration through the skin. The main method by which topically applied surfactant nanoparticles promotes transdermal medication penetration is owing to the extraction of epidermal lipids or the disruption of the organized arrangement of corneocytes after bonding to the keratin strand [49]. In this study, the formulation containing surfactant would have also worked as a chemical enhancer to assist the transport of the formulation through the skin as described elsewhere [54]. 

### 2.12. In Vivo Pharmacokinetic Studies

**Figure 12:**
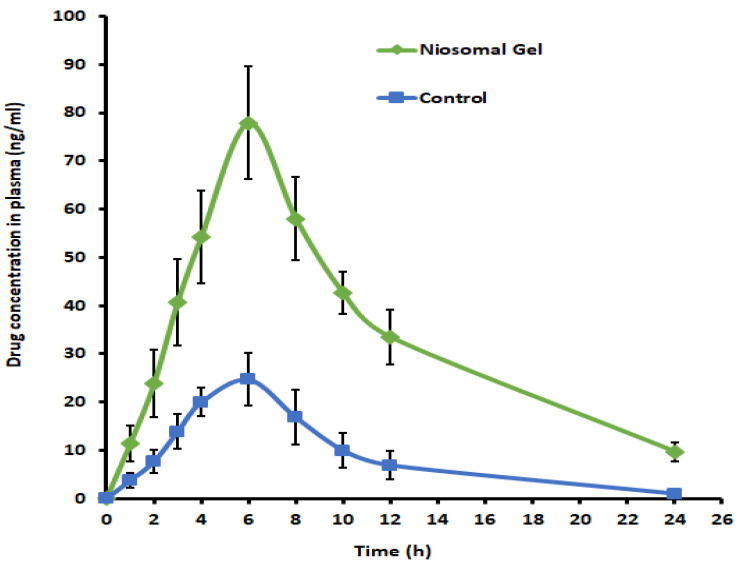
Plasma drug concentrations–time profiles of levosulpiride from niosomal gel and control gel in rats. Each value depicts the average and standard deviation of six different batches.

In vivo evaluation was conducted in Wistar rats to examine the pharmacokinetics of levosulpiride from drug-loaded niosomes and control gel, which also could provide evidence about its potential for clinical use. Figure 12 compares the levosulpiride mean plasma concentration profile of niosomal gel and control in rats. In Table 3, various pharmacokinetic parameters including AUC_0-α_, C_max_, and T_max_ derived from the non-compartment analysis [55] are compiled. Figure 12 signifies that the levosulpiride plasma profile of the niosomal gel was more prominent (*p < 0*.005) as compared to the control. In both cases, the drug absorption was relatively low in the initial hour, though it increased with duration. The plasma drug level reached a maximum at 6 h in both cases, however, the levosulpiride level was significantly higher (~3 folds) with niosomes (*p* < 0.0001) in comparison to the control gel (Table 3). The greater C_max_ values observed with the niosomal gel signify the potential of optimized formulation to transport a higher level of levosulpiride across the skin barrier. Followed by the C_max_, the drug level decreases slowly during the study period. On the other hand, a higher rate and a higher extent of drug absorption (bioavailability) were evidenced by greater AUC_0-α_ (ng.h/mL) values in niosomal gel (~500% higher; *p* < 0.0001) than its counterpart, an agreement with the ex vivo permeation study (Figure 11). The low drug absorption from the control gel could be correlated to the levosulpiride’s low solubility and permeability (BCS class IV). The AUC_0-α_ data observed in this investigation is comparable to the intramuscular administration in humans (1724 ng.h/mL) [25] and less than using the microneedle patch (986 µg.h/mL) with a higher dose (25 mg) in mice [56]. Overall, the data here signified that the transdermal therapy of levosulpiride using niosomes as a carrier could be a promising approach.

### 2.13. Stability Studies

The results of various parameters examined (viscosity, pH, drug content, and spreadability) during the stability study of prepared levosulpiride-loaded niosomal gel indicate no significant variation in the parameters tested (Table 4). The in vitro drug release profile (Figure S2) after three months of stability was studied by t-test with two samples having identical variances. After the designated stability period, the optimized gel showed a statistically negligible difference, as indicated by the obtained t-test value of 0.209, which was significantly lower than the t-critical value of 1.70. Overall, the data here signifies that the optimized formulation did not show any significant variations in all the parameters tested during 3 months when stored at 25 °C. 

## 3. Conclusions

This study evaluated the possibility of niosomes to improve the transdermal therapy of levosulpiride, which has limited aqueous solubility and low permeability. Preliminary trials were performed to choose the surfactant, and formulation optimization was performed using the Box-Behnken design. Cholesterol content showed a positive effect, while Span 40 and sonication time showed a negative effect on both dependent variables (niosome size and % EE). Optimized niosomes (NC) vesicles exhibited all physicochemical properties within satisfactory limits for transdermal delivery. The FTIR and DSC data show that the drug and the other excipients utilized are compatible. Levosulpiride release (*p* < 0.01) from the drug-loaded niosomes gel and permeation (*p* < 0.01) through the skin was found significantly higher, as compared to the control. AUC measurements of the relative bioavailability of levosulpiride revealed a 5-fold greater concentration in plasma from prepared niosomes vesicles than the control. The stability assessment indicates that the optimized levosulpiride-loaded niosomes have enough physical as well as chemical stability for three months. This study confirms the possible utility and practical viability of developed niosomes gel for transdermal therapy of levosulpiride.

## 4. Materials and Methods

### 4.1. Materials

Levosulpiride was obtained from Zydus Healthcare Ltd. (Ahmedabad, India). Span (20, 40, 60, 80), Tween (20, 40, 60, 80), methanol, chloroform, and Carbopol 934 were purchased from Chemdyes Corporation (Ahmedabad, India). Cholesterol was obtained as a gift from Emcure Pharmaceuticals Ltd. (Ahmedabad, India).

### 4.2. Preliminary Trials for Selection of Excipients

Preliminary batches of niosomes (P1-P24) were prepared with 50 mg of levosulpiride, surfactants (Spans and Tweens), and cholesterol in a molar ratio of 1:1 and different sonication time (2, 10, and 18 min) and assessed for entrapment efficiency (% EE). Based on preliminary trials (Table S1), it was found that the % EE was highly affected by the sonication time. 

### 4.3. Formulation of Levosulpiride-Loaded Niosomes

Niosomes containing levosulpiride were formulated by the thin film hydration method. Briefly, 50 mg of levosulpiride, cholesterol, and Span 40 were precisely weighed and a mixture of methanol:chloroform (1:1; 10 mL) was added to dissolve them. The whole mixture was added to a rotary evaporator and the organic solvent was separated at 60 °C by applying a vacuum. The removal of solvent led to the formation of a thin film, which was dried and further hydrated with phosphate buffer (pH 7.4; 10 mL) for 10 min with continuous stirring (100 rpm) using a magnetic stirrer (Remi Sales & Engineering Ltd., Mumbai, India). The nanovesicles obtained were cooled and sonicated using a probe sonicator (Vibra Cell Ultrasonic Processor, VC 750, M/s. Sonics and Materials Inc., Newtown, CT, USA) with power (230 V, 50 Hz) to obtain levosulpiride-loaded niosomes.

The preparation was subjected to freeze-drying by homogenizing 5% mannitol (cryoprotectant) with 10 mL of the formulation [57]. The formulation was then transferred to a 15 mL freeze-drying vial and placed in a deep freezer (RQVD-300 PLUS, Remi Elektrotechnik Ltd., Mumbai, India) at −20 °C for 24 h. Lyophilization was performed using a freeze dryer (TFD8503, ilShinBioBase Co. Ltd., Gyeonggido, South Korea) at −80 °C for 36 h [58]. Finally, to eliminate any residual moisture, the freeze-dried formulation was subjected to air-drying at 25 °C for 1 h.

### 4.4. Estimation of Levosulpiride

HPLC system (Shimadzu, Tokyo, Japan) with a photodiode array detector was selected to estimate the presence of levosulpiride in various samples. Levosulpiride was separated chromatographically using a Phenomenex C-18 column (150 × 4.6 mm, i.d 5 μm), phosphate buffer (10 mM), and acetonitrile (85:15) as eluent. Isocratic elution of the drug was obtained by letting the eluent pass through the column (25 °C) at a particular flow rate (1 mL/min). The volume of the sample injected was 50 µL, while the eluate was monitored at 250 nm. The retention time was recorded at 3.2 min. Sulfamethoxazole was used as an internal standard. The regression analysis showed satisfactory linearity (r^2^ = 0.992) when the concentration of levosulpiride was between 5 and 600 ng/mL.

### 4.5. Optimization Study of Variables 

The selection of experimental design is important and also influenced by the number of variables. Indeed, when the number of variables increases the model equations become more complex. Box Behnken design is specially designed for a second-order regression model (quadratic model) having different variables and levels. Moreover, it provides an efficient estimation of the coefficients, is rotatable, and needs fewer experimental runs. This design is an independent quadratic design and considers the midpoints of the edges of the factors and the centre point in the process space. It also estimates the curvature- interactions of the variables [59]. Hence, the prepared levosulpiride-loaded niosomes were optimized by Box-Behnken design using Design Expert (Version 11, Minneapolis, MN) to assess the main, interactive, and quadratic impacts [60]. The input variables chosen for the study were cholesterol (X_1_, mM), Span 40 (X_2_, mM), and sonication time (X_3_, min) with their three levels. The responses selected were the size of the niosomes particle (Y_1_, nm) and EE (Y_2_, %) as per Table 5. According to the design, a total of fifteen experimental runs (N1-N15) were performed (Table 6). The equation used for the nonlinear quadratic model is shown below:

Y_1_ = b_0_ + b_1_X_1_ + b_2_X_2_ + b_3_X_3_ + b_12_X_1_X_2_ + b_13_X_1_X_3_ + b_23_X_2_X_3_ + b_11_X_1_^2^ + b_22_X_2_^2^ + b_33_X_3_^2^

where Yi is the input variable, b_0_ is the intercept, and b_1_ to b_33_ are estimated partial regression coefficients. The interaction and quadratic terms are denoted by the terms X_1_X_2_ and Xi^2^ (i = 1, 2, and 3), respectively. The check point batch was formulated to validate the model and 3D surface and contour plots were plotted in estimating the impact of variables on responses.

### 4.6. Characterization of Levosulpiride-Loaded Niosomes 

#### 4.6.1. Particle Size and Zeta Potential

A Zetasizer (Horiba SZ-100, Kyoto, Japan) was used to measure the particle size (hydrodynamic diameter) distribution, polydispersity index, and zeta potential of the prepared niosomes using a dynamic light scattering technique. A precisely weighed amount of niosomes (1 mL) was dispersed in water (50 mL) and kept on a glass cuvette to measure the particle size at 90°, while the temperature was maintained at 25 °C [61]. The zeta potential of prepared niosomes was measured under the electric potential of 25 V/cm to vibrate the charged niosomes [62]. The data presented are the average of three batches.

#### 4.6.2. Entrapment Efficiency

The % EE of levosulpiride in prepared niosomes was estimated by the centrifugation method described in the literature [63,64]. Formulation (200 mg) was taken in a glass vial, to which was added 10 mL of PBS solution (pH 7.4), and dispersed by vortexing for 10 min. The mixture was centrifuged at 12,298× *g* at 4 °C for 30 min to separate the free drug. This separation procedure was repeated thrice and confirmed the complete removal of surface drug particles. The supernatant was collected (30 mL), 0.1 mL of this solution was diluted to 100 mL using mobile phase, and the amount of levosulpiride was estimated using HPLC as mentioned before. The data presented are the average of three batches. The % EE of niosomes formulations was determined using the following equation.

% EE = (Total drug added − Drug in the supernatant)/(Total drug added) × 100

### 4.7. Formulation of Check Point Batch

To assess the validity of the design model, a check point batch (NC) was selected. After analysis of all parameters, an overlay plot was developed in design expert software. The overlay plot marked by the yellow region may provide the desired result for all parameters. From the overlay plot, by point prediction, a point was selected in the yellow region to get predicted values of independent and estimated values of responses. The formulation layout and composition of drugs and excipients in check point batch are depicted in Table 7 and Table 8.

The software suggested the optimized formulation (check point batch based on the overlay plot) with 0.99 desirabilities by considering X_1_ = 309.32 mg (Cholesterol), X_2_ = 198.07 mg (Span 40), and X_3_ = 10 min (sonication time) to achieve Y_1_ and Y_2_ results (Theoretical and practically) shown in Table 7 and Table 8. This optimized batch was used to evaluate other parameters like polydispersity index and zeta potential. 

### 4.8. Fourier Transform Infrared (FTIR) 

FTIR spectra of drug, cholesterol, physical mixture, and niosomes were recorded in the range of 400 to 4000 cm^−1^ using a spectrophotometer (FT/IR-6100, Jasco, Tokyo, Japan). For the FTIR analysis, a small quantity of samples was added to the KBr powder and grind them together using mortar and pestle. The mixture was transferred to the stainless-steel sample holder and recorded the spectra.

### 4.9. Differential Scanning Calorimetry (DSC)

DSC analysis of the drug, cholesterol, physical mixture, and niosomes were carried out using a DSC instrument (DSC 7020, Hitachi, Tokyo, Japan). A precisely weighed (5 mg) test sample was kept in an aluminum pan and an empty pan as a reference for the analysis. The thermal behavior of samples was measured between 30–300 °C at a 10 °C/min heating rate in a nitrogen atmosphere.

### 4.10. Transmission Electron Microscopy (TEM)

The structural features of niosomes were determined using TEM (Tecnai 20, Philips, The Netherlands). The instrument was run at 200 kV and had 0.15 nm efficiency and a high resolution. TEM study was performed using phosphotungstic acid (2%) to stain niosomes and place them on the copper grids, before being dried at room temperature (25 °C).

### 4.11. Preparations of Levosulpiride Niosomal Gel

The optimized niosomes containing levosulpiride were transformed into a suitable gel to have greater skin retention for a longer duration. Briefly, carbopol gel composition was prepared by mixing Carbopol 934 (1 g) with water (up to 100 g) to obtain 1% *w*/*w* and kept for 12 h for complete wetting of the polymer. Optimized niosomes (NC) gel was prepared by weighing niosomes equivalent to 50 mg of levosulpiride and added to the above-prepared carbopol solution (~9 g). To this mixture, triethanolamine (0.1% *w*/*w*) was added slowly and stirred to obtain a viscous composition. The final volume of gel (10 g) was adjusted by adding carbopol solution to get optimized levosulpiride niosomes gel [65]. Control gel was prepared by adding levosulpiride solution (dissolved in ethanol) equivalent to 50 mg to overnight hydrated Carbopol (1% *w*/*w*) solution containing Span 40 (3% *w*/*v*), and the homogenous gel was obtained by including triethanolamine (0.1% *w*/*w*).

### 4.12. Evaluation of Levosulpiride-Niosomal Gel

#### 4.12.1. Viscosity

A Brookfield Synchro-Lectric viscometer (LVDVI prime, Middleborough, MA, USA) was used to assess the viscosity of gel formulation using spindle #95 at 50 rpm at 25 ± 1.0 °C or 32 ± 1.0 °C. The data presented are the average of three different batches.

#### 4.12.2. pH

The pH was recorded using a Mettler Toledo pH meter (MP-220, Greifensee, Switzerland) after inserting the electrode in a gel formulation and equilibrating it for 1 min. The data presented are the average of three batches.

#### 4.12.3. Drug Content

Weighed gel (1 g) was taken in propylene vials, mixed with methanol (10 mL), and sonicated for 30 min at 25 °C. After this, the mixture was centrifuged at 12,000 rpm for 20 min and the supernatant was taken and filtered through a membrane filter (0.2 μm, pore size). The filtered solution (0.1 mL) was subsequently diluted with mobile phase (100 mL) and the drug content was estimated using HPLC. The data presented are the average of three different batches.

#### 4.12.4. Spreadability

The spreadability of gel formulation was carried out by placing 0.5 g in a glass plate (5 cm in diameter), and the second glass plate was placed on it and kept aside (5 min) without disturbing it to confirm there had been no further spreading of formulation. The diameters of the spread formulation were measured by marking the circles. The data presented are the average of three different batches. 

#### 4.12.5. Morphology

An optical microscope (CX21iLED, Olympus, Hamburg, Germany) was used for evaluating the morphology of the prepared gel. A small quantity of the gel was mounted on a glass slide (76 × 26 × 1 mm) and the image was captured with a 40× objective and 10× ocular lens [23]. Images were visualized with the aid of image view software.

#### 4.12.6. Mechanical Properties

The mechanical properties of the optimized gel were tested by measuring adhesiveness and hardness by following standard procedure [66]. Both the properties of gel were evaluated by employing a texture analyzer (QTS-25, Brookfield Engineering Labs, Middleboro, MA, USA) using software Texture Pro 2.1 v. Gel was filled in the cylindrical sample holder (35 mm diameter and 45 mm height) of the texture analyzer, coupled with a 5-kg load cell. Then, the probe was immersed in the gel at a speed of 30 mm/min until 20 mm distance had been traveled by the probe. After that probe was pulled up at the same speed and the adhesiveness and hardness of the gel were measured at which the gel completely detaches.

### 4.13. In Vitro Release Study

The drug release from the prepared niosomes was carried out using a vertical Franz diffusion setup recommended for the release test of topical formulations [67]. Cellulose dialysis membrane (2.4 nm pore size; Himedia, Mumbai, India) [68] soaked overnight in water was used as a release barrier for the formulations tested. The membrane was mounted between the receiver and donor compartment and the active area available for drug release was 1.13 cm^2^. Accurately weighed gel formulation (0.5 g containing 2.5 mg of levosulpiride) was placed in the donor chamber and covered with Parafilm [69]. The release media [10 mL of phosphate buffer (pH 7.4) with Tween 80 (10% *w*/*v*)] was added to the receiver chamber, the temperature was set at 32 ± 0.5 °C, and was stirred at 50 rpm. Samples (2 mL) were collected from the receptor compartment at specific time points and an equal volume was replaced with fresh media. The samples were filtered, diluted with mobile phase, and analyzed by HPLC. For comparison, a similar release study of the control gel [0.5 g containing 2.5 mg of levosulpiride in Carbopol (1% *w*/*w*) and Span 40 (3% *w*/*v*), neutralized with triethanolamine (0.1% *w*/*w*)] was performed. The data presented are the average of six batches.

### 4.14. Ex Vivo Permeation Study

The penetration potential of levosulpiride from both optimized formulation and control formulation was carried out using full-thickness rat skin in a Franz diffusion cell setup, as mentioned in the in vitro release study section. The skin membrane surface was applied with gel formulation (0.5 g containing 2.5 mg levosulpiride) or control gel [70]. At specific time intervals (1, 2, 4, 6, 8, 10, 12, 18, and 24 h), samples were withdrawn and analyzed by HPLC. Various permeation parameters were determined using the formula mentioned in the literature [71]. The data presented is the average of six batches.

### 4.15. In Vivo Pharmacokinetic Studies

The in vivo permeation potential of levosulpiride-loaded niosomes was evaluated in adult male Wistar rats having weights around 200–250 g (aged 6–8 weeks). Animals were kept in the animal house for 24 h while being watched, and were given a regular pellet diet along with unlimited access to water. The use of animals in this research was according to the institutional policies and procedures that were approved in the ethical approval (ASPBRI/IAEC/2021-22/05; dated 2 March 2022). Rats were distributed in two groups, each group having six rats. Phenobarbitone (30 mg/kg; intraperitoneal) was used to anesthetize the animals, and an electric clipper was used to precisely shave the dorsal hair. The application site was cleaned with phosphate buffer saline and dried using cotton swabs. A custom-made open-ended holder with wings (applicator, with a diameter of 1 cm) was fixed on the skin surface. Group 1 rats were applied with one gram of the drug-loaded niosomal gel (containing 5 mg of levosulpiride). Group 2 animals were applied with the control gel containing the same amount of drug (5 mg), dispersed in 1 g of carbopol (1% *w*/*w*) and Span 40 (3% *w*/*v*). The gel was uniformly spread on the skin surface (1 cm^2^) and covered with Parafilm. The animal dose of levosulpiride was determined using a formula mentioned in the literature [72] by considering the recommended human dose (300 mg). At 1, 2, 4, 6, 8, 12, and 24 h, 200 µL of blood was withdrawn from the lateral tail vein. Rats were administered 250 µL of dextrose (intraperitoneal injection) after each blood sample to reduce the sharp variations in central compartmental volume. Plasma samples were mixed with the same volume of acetonitrile and 2-propanol (1:1), and then vortexed (2 min) to precipitate proteins. The samples were further centrifuged (1789× *g* for 15 min) and assayed by HPLC. The in vivo parameters were determined by noncompartmental pharmacokinetic analysis. C_max_ (peak plasma concentration) and T_max_ (time to reach C_max_), values were measured from each plasma profile, while the linear trapezoidal rule was used to determine AUC_0-α_ (the total area from zero to infinity). The average value and standard deviation of six trials are presented. 

### 4.16. Stability Studies

The stability of the developed levosulpiride-loaded niosomal gel was tested for three months at a controlled room temperature of 25 ± 2 °C and relative humidity of 60 ± 5% based on the ICH guidelines [73,74]. Samples were preserved in a stability cabinet and housed in light-resistant screw-capped glass vials. The drug content, pH, spreadability, and viscosity were evaluated. The drug release study was carried out using a vertical Franz diffusion setup as described in the in vitro release study section. 

### 4.17. Statistical Analysis

GraphPad Prism (version 6, San Diego, CA, USA) was used to analyze statistical variances between trials. A difference in values of *p* < 0.05 was used to define statistical significance.

## Figures and Tables

**Table 1:** Composition and parameters evaluated for Box-Behnken design batches. The data presented are average ± SD of different batches (n = 6).

Batches	X_1_; Cholesterol (mM)	X_2_; Span 40 (mM)	X_3_; Sonication Time (min)	Y_1_; Particle Size (nm)	Y_2_; EE (%)
N1	1	0.5	10	77.3 ± 8.7	65.24 ± 1.51
N2	3	0.5	10	202.6 ± 26.3	88.34 ± 3.51
N3	1	1.5	10	72.4 ± 9.4	51.76 ± 1.36
N4	3	1.5	10	194.3 ± 15.2	86.21 ± 2.26
N5	1	1.0	5	77.6 ± 10.5	59.13 ± 1.29
N6	3	1.0	5	201.9 ± 22.1	88.54 ± 2.38
N7	1	1.0	15	73.8 ± 6.9	59.92 ± 1.62
N8	3	1.0	15	195.2 ± 18.5	88.76 ± 2.57
N9	2	0.5	5	147.8 ± 16.6	80.43 ± 3.11
N10	2	1.5	5	144.4 ± 14.7	70.54 ± 2.71
N11	2	0.5	15	142.5 ± 12.1	80.32 ± 3.35
N12	2	1.5	15	139.1 ± 14.3	69.12 ± 1.84
N13	2	1.0	10	198.5 ± 20.5	74.34 ± 2.04
N14	2	1.0	10	199.7 ± 16.4	75.67 ± 2.20
N15	2	1.0	10	200.3 ± 22.5	73.45 ± 1.94

**Table 2:** Estimated and observed values for check point analysis of optimized batch (NC).

Particle Size (nm)		EE (%)		Polydispersity Index	Zeta Potential (mV)
Estimated Value	Observed Value	Estimated Value	Observed Value		
102.3	102.2	68.537	67.98	0.218	−49.9

**Table 3:** Estimated pharmacokinetic parameters of levosulpiride in plasma after transdermal administration in Wistar rats. Each value depicts the average and standard deviation of six different batches.

Parameters	Niosomal Gel	Control
*T*_max_ (h)	6	6
*C*_max_ (ng/mL)	77.94 ± 11.66 *	24.71 ± 5.48
*AUC*_0-α_ (ng.h/mL)	1083.43 ± 252.33 *	223.51 ± 49.65

* *p* < 0.05 were considered significant in comparison to the control.

**Table 4:** Stability data of optimized levosulpiride-loaded niosomal gel. Each value depicts the average and standard deviation of different batches (n = 3).

Evaluation Parameters	Initial	After Three Months
Viscosity (cPs)	1740 ± 75	1760 ±60
pH	6.9 ± 0.13	6.8 ± 0.19
Drug content (%)	97.20± 2.13	96.44 ± 2.69
Spreadability (cm)	4.6 ± 0.22	4.3 ± 0.31

**Table 5:** Factors and response variables for Box-Behnken design.

Factors	Levels		
	−1	0	1
X_1_ = Cholesterol (mM)	1	2	3
X_2_ = Span 40 (mM)	0.5	1	1.5
X_3_ = Sonication time (min)	5	10	15
Responses	Y_1_ = Particle size (nm) Y_2_ = EE (%)		

**Table 6:** Formulation layout of design batches.

Batches	Coded Values			Actual Values		
	X_1_	X_2_	X_3_	X_1_ (mM)	X_2_ (mM)	X_3_ (min)
N1	−1	−1	0	1	0.5	10
N2	+1	−1	0	3	0.5	10
N3	−1	+1	0	1	1.5	10
N4	+1	+1	0	3	1.5	10
N5	−1	0	−1	1	1.0	5
N6	+1	0	−1	3	1.0	5
N7	−1	0	+1	1	1.0	15
N8	+1	0	+1	3	1.0	15
N9	0	−1	−1	2	0.5	5
N10	0	+1	−1	2	1.5	5
N11	0	−1	+1	2	0.5	15
N12	0	+1	+1	2	1.5	15
N13	0	0	0	2	1.0	10
N14	0	0	0	2	1.0	10
N15	0	0	0	2	1.0	10

**Table 7:** Formulation layout for check point batch (NC).

Coded Value			Actual Value		
X_1_	X_2_	X_3_	X_1_; Cholesterol (mM)	X_2_; Span 40 (mM)	X_3_; Sonication time (min)
−0.800	−0.985	0	0.800	0.492	10

**Table 8:** Composition of drug and excipients in check point batch (NC).

Levosulpiride (mg)	X_1_; Cholesterol (mg)	X_2_; Span 40 (mg)	X_3_; Sonication Time (min)	Chloroform (mL)	Methanol (mL)
50	309.32	198.07	10	10	10

## Data Availability

The data presented in this study are contained within the article.

## Supplementary Materials

The following supporting information can be downloaded at: https://www.mdpi.com/article/10.3390/gels9030213/s1, Table S1. Preliminary trials (P1-P24) used for the selection of non-ionic surfactants in preparing Levosulpiride-loaded niosomes. Table S2. ANOVA for the quadratic model for particle size. Table S3. ANOVA for the quadratic model for entrapment efficiency. Figure S1. Load versus time curve of hardness/adhesiveness force of niosomal gel. Figure S2. In vitro release profile of Levosulpiride from niosomal gel after three months of stability study.
