# Supplementary material for: Qbd-Based Approach to Optimize Niosomal Gel of Levosulpiride for Transdermal Drug Delivery
Source: Gels. 2023 Mar 10;9(3):213. doi: 10.3390/gels9030213 (PMC10048649; doi:10.3390/gels9030213)
Supplement: Supplementary file 1 [file gels-09-00213-s001.zip › gels-2265038-supplementary.pdf]

gels

Supplementary Materials

Qbd-Based Approach to Optimize Niosomal Gel of Levosulpiride for Transdermal Drug Delivery

Ahmed S. Alnaim [1,*,†], Hiral Shah [2,†], Anroop B. Nair [1], Vivek Mewada [3], Smit Patel [2], Shery Jacob [4], Bandar Aldhubiab [1], Mohamed A. Morsy [1,5], Rashed M. Almuqbil [1], Pottathil Shinu [6] and Jigar Shah [4,*]

Table S1. Preliminary trials (P1-P24) used for the selection of non-ionic surfactants in preparing Levosulpiride*-loaded niosomes.

Batches	Non-ionic surfactants	Surfactant: cholesterol (molar ratio)	Sonication time (min)	% entrapment efficiency
P1	Span 20	1:1	2	56.34
P2	Span 20	1:1	10	58.71
P3	Span 20	1:1	18	53.67
P4	Span 40	1:1	2	61.23
P5	Span 40	1:1	10	64.21
P6	Span 40	1:1	18	60.34
P7	Span 60	1:1	2	57.34
P8	Span 60	1:1	10	58.76
P9	Span 60	1:1	18	54.33
P10	Span 80	1:1	2	54.45
P11	Span 80	1:1	10	58.41
P12	Span 80	1:1	18	52.23
P13	Tween 20	1:1	2	53.34
P14	Tween 20	1:1	10	55.71
P15	Tween 20	1:1	18	52.67
P16	Tween 40	1:1	2	55.23
P17	Tween 40	1:1	10	56.21
P18	Tween 40	1:1	18	53.34
P19	Tween 60	1:1	2	56.34
P20	Tween 60	1:1	10	58.76
P21	Tween 60	1:1	18	53.33
P22	Tween 80	1:1	2	54.45
P23	Tween 80	1:1	10	58.53
P24	Tween 80	1:1	18	51.11

*The drug used is 50 mg.

Table S2. ANOVA for the quadratic model for particle size.

Source	Sum of Squares	df	Mean Square	F-value	p-value	
Model	39357.57	9	4373.06	3087.23	< 0.0001	significant
A-Cholesterol	30368.80	1	30368.80	21439.32	< 0.0001	
B-Span 40	50.00	1	50.00	35.30	0.0019	
C-Sonication time	55.65	1	55.65	39.29	0.0015	
AB	2.89	1	2.89	2.04	0.2126	
AC	2.10	1	2.10	1.48	0.2774	
BC	0.0000	1	0.0000	0.0000	1.0000	
A^2	4417.09	1	4417.09	3118.31	< 0.0001	
B^2	2949.30	1	2949.30	2082.10	< 0.0001	
C^2	2851.00	1	2851.00	2012.71	< 0.0001	

Table S3. ANOVA for the quadratic model for entrapment efficiency.

Source	Sum of Squares	df	Mean Square	F-value	p-value	
**Model**	1887.66	9	209.74	147.33	< 0.0001	significant
A-Cholesterol	1676.20	1	1676.20	1177.45	< 0.0001	
B-Span 40	168.36	1	168.36	118.26	0.0001	
C-Sonication time	0.0338	1	0.0338	0.0237	0.8836	
AB	32.21	1	32.21	22.62	0.0051	
AC	0.0812	1	0.0812	0.0571	0.8207	
BC	0.4290	1	0.4290	0.3014	0.6066	
A^2	6.31	1	6.31	4.43	0.0892	
B^2	0.3150	1	0.3150	0.2213	0.6579	
C^2	3.04	1	3.04	2.14	0.2035	

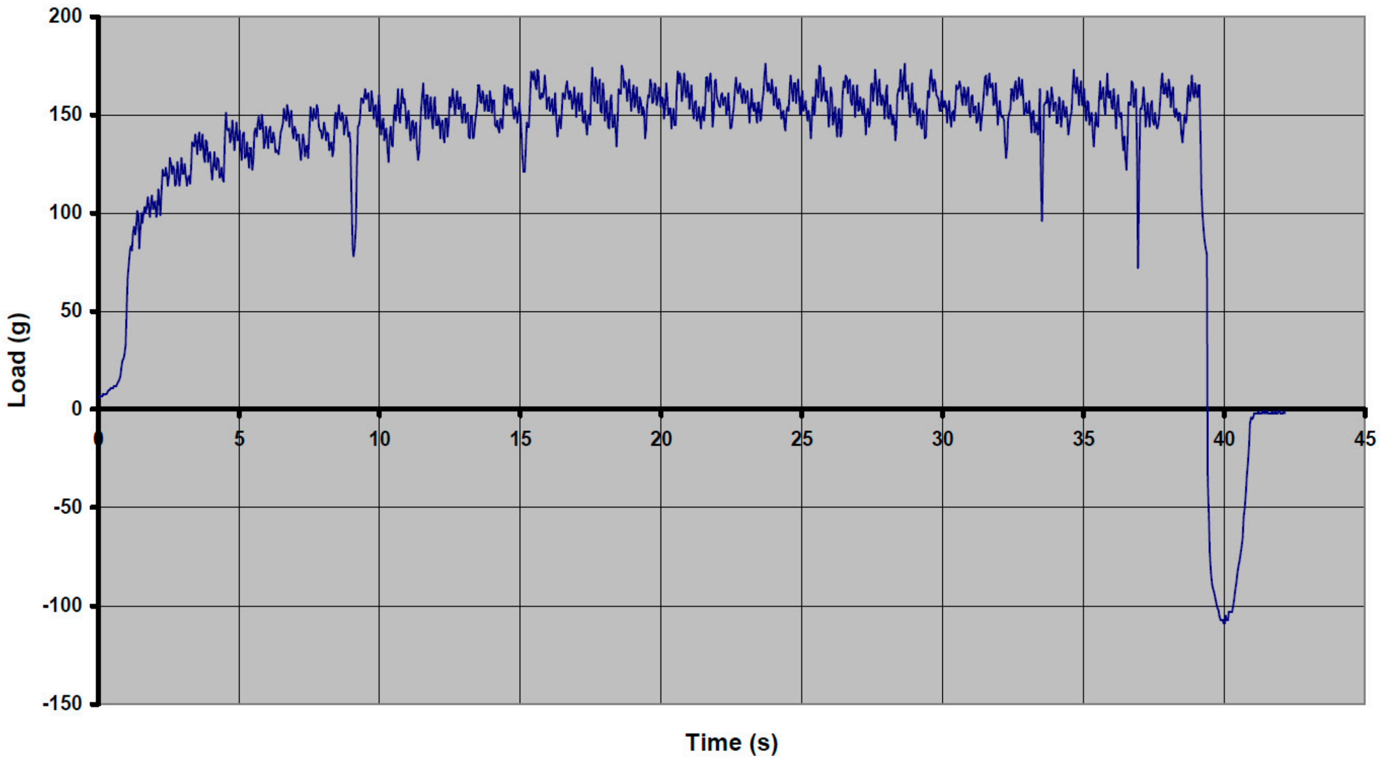

Figure S1. Load versus time curve of hardness/adhesiveness force of niosomal gel.

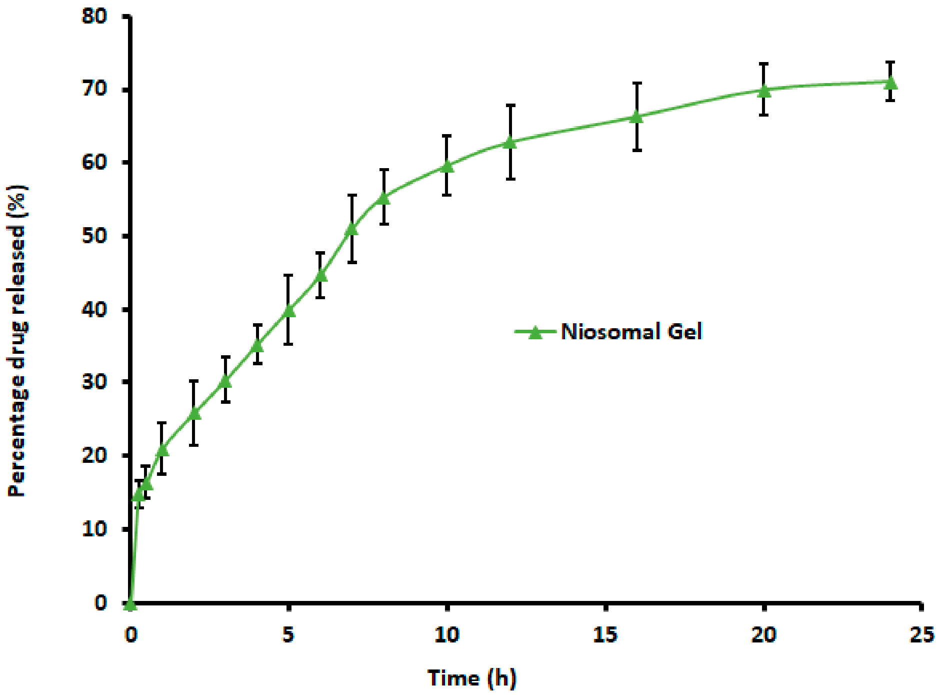

Figure S2. In vitro release profile of Levosulpiride from niosomal gel after three months of stability study.
